# An Artificial Neural Network Prediction Model of Depressive Symptoms among Women with Abnormal Papanicolaou Smear Results before and after Diagnostic Procedures

**DOI:** 10.3390/life14091130

**Published:** 2024-09-07

**Authors:** Irena Ilic, Goran Babic, Aleksandra Dimitrijevic, Sandra Sipetic Grujicic, Milena Ilic

**Affiliations:** 1 Faculty of Medicine, University of Belgrade, 11000 Belgrade, Serbia; 2 Department of Gynecology and Obstetrics, Faculty of Medical Sciences, University of Kragujevac, 34000 Kragujevac, Serbia; 3 Institute of Epidemiology, Faculty of Medicine, University of Belgrade, 11000 Belgrade, Serbia; 4 Department of Epidemiology, Faculty of Medical Sciences, University of Kragujevac, 34000 Kragujevac, Serbia

**Keywords:** artificial neural networks, depression, cervical cancer screening, Papanicolaou smear, diagnostic procedures

## Abstract

(1) Background: Cervical screening and additional diagnostic procedures often lead to depression. This research aimed to develop a prediction model for depression in women who received an abnormal Papanicolaou screening test, prior to and following the diagnostic procedures. (2) Methods: The study included women who had a positive Papanicolaou screening test (N = 172) and attended the Clinical Center of Kragujevac in Serbia for additional diagnostic procedures (colposcopy/biopsy/endocervical curettage). Women filled out a sociodemographic survey and the Center for Epidemiologic Studies Depression questionnaire (CES-D scale) before and after diagnostic procedures. A prediction model was built with multilayer perceptron neural networks. (3) Results: A correlation-based filter method of feature selection indicated four variables that correlated with depression both prior to and following the diagnostic procedures—anxiety, depression, worry, and concern about health consequences. In addition, the use of sedatives and a history of both induced and spontaneous abortion correlated with pre-diagnostic depression. Important attributes for predicting post-diagnostic depression were scores for the domains ‘Tension/discomfort’ and ‘Embarrassment’ and depression in personal medical history. The accuracy of the pre-diagnostic procedures model was 70.6%, and the area under the receiver operating characteristic curve (AUROC) was 0.668. The model for post-diagnostic depression prediction showed an accuracy of 70.6%, and an AUROC = 0.836. (4) Conclusions: This study helps provide means to predict the occurrence of depression in women with an abnormal Papanicolaou screening result prior to and following diagnostic procedures, which can aid healthcare professionals in successfully providing timely psychological support to those women who are referred to further diagnostics.

## 1. Introduction

According to GLOBOCAN 2022 estimates, cervical cancer ranked as the fourth most common cancer in women in Serbia (with about 900 new cases), and the fifth most common cancer in terms of mortality (with about 500 deaths) [1]. During the last few decades in developed countries, the implementation of the screening program based on the use of the Papanicolaou test has led to a significant decline in incidence and mortality from cervical cancer [2,3,4]. The implementation of organized, decentralized cervical cancer screening in Serbia began in 2013, and the basic test used for screening is the cytological Papanicolaou (Pap) smear of the cervix [5].

An abnormal Pap test result includes atypical squamous cells of undetermined significance (ASC-US), atypical glandular cells (AGC), low-grade squamous intraepithelial lesions (LSIL), high-grade squamous intraepithelial lesions (HSIL), adenocarcinoma in situ (AIS), and cervical cancer cells (squamous cell carcinoma or adenocarcinoma) [6]. Unfortunately, not many population-based epidemiological studies on cervical intraepithelial neoplasia have been published. During 2016, a total of 76,609 women in the target group (aged 25–64 years) were tested (had Papanicolaou test) in Serbia, and among them 665 (0.87%) were with premalignant (intraepithelial) lesions, while 28 (0.04%) were with invasive carcinoma [7]. For every 100,000 women in the United States, eight new cervical cancer cases and two deaths were reported in 2017 [8]. Based on nationwide registries in Denmark between 1997 and 2012, the incidences of AIS increased significantly in middle-aged women, but they decreased significantly in women aged ≤20 years, coinciding with the introduction of free-of-charge human papillomavirus vaccination [9].

However, a certain number of women who receive positive Pap test results do not adhere to the recommended guidelines and do not go through further diagnostic procedures [10,11]. Reasons for non-adherence to follow-up diagnostics after an abnormal Pap result, sometimes include increased levels of depression [12,13].

Research has shown that the frequency of depression after colposcopy ranged from 7% to 22% [14,15,16]. The results of most studies indicated that depression was lower after colposcopy [16,17,18]. In a study in Great Britain [14], the frequency of depression (defined as a score of ≥8 according to the “Hospital Anxiety and Depression Scale” (HADS) questionnaire—depression subscale) was lower after (6.6%) than before colposcopy (7.9%). A two-year follow-up study of depression in women who were referred to colposcopy after receipt of an abnormal cervical smear within the organized screening in some countries showed that the level of depression decreased over time [19,20]. This study aimed to develop artificial neural network models for predicting depression prior to and following diagnostic procedures in women who received a positive Pap screening test.

## 2. Methodology

### 2.1. Setting

This study was conducted at the Clinic of Gynecology and Obstetrics at the Clinical Center Kragujevac, a large University hospital in Serbia, where women who had received a positive cervical smear within the screening program were referred to, within four to six weeks, for diagnostics (consultative colposcopy/biopsy/endocervical curettage).

### 2.2. Study Design

In this research, a cross-sectional study design was used in a cohort of women who had received abnormal Pap screening smear results.

### 2.3. Study Sample

All women who attended cervical cancer screening and received an abnormal Pap smear result and then underwent further diagnostic examination were included in the sample. Women were eligible if they were 20–65 years old, had a Pap smear taken routinely within the population screening program that showed an abnormality in the previous 12 months, were residents of the Kragujevac district area, and were fluent in spoken and written Serbian language.

Women were not eligible for participation if they were <20 and >65 years old, were pregnant at recruitment, or were previously treated for cervical lesions. The exclusion criteria for subjects involved reproductive organ disease the treatment of which was ongoing during the study, refusing to participate, or presence of any other objective reason that hindered participation.

### 2.4. Sample Size Calculation

According to Sharp and coauthors [14], women with a positive Pap screening smear had a prevalence of depression (according to the HADS, i.e., score ≥ 8 on the HADS depression subscale), of 7.9% prior to and 16.0% following the diagnostic procedures. Using the software Epi Info Version 7.2.0.1 (Centers for Disease Control and Prevention, Atlanta, Georgia) and Fleiss’s formula with continuity correction (first type error α of 0.05 and desired study strength of 95%), it was determined that a minimum sample of 154 subjects was required.

### 2.5. Data Collection

Women who were eligible to participate in the study were asked to fill out a sociodemographic survey and the Center for Epidemiologic Studies Depression questionnaire (CES-D scale) [21]. All participants provided written informed voluntary consent prior to taking part in the study. They had approximately 20 (±5) min to complete the survey. The respondents filled out the questionnaires immediately prior to having diagnostic procedures carried out (1st study time point) and immediately prior to receiving the results of the performed procedures, after a period of 2–4 weeks (2nd study time point).

Refusal to participate was documented. Out of the 238 eligible women, the study sample included a total of 172 women (participation rate: 72.3%). Questionnaires that were not fully completed were not included in the analysis.

### 2.6. Instruments

A demographic questionnaire was used to obtain information about participants’ age (≤30/31–40/41–50/51–60/≥61), place of residence (Rural/Urban), education level (≤8 years/>8 years), and marital status (Without partner/With partner).

In addition, questionnaires CES-D [21], the “Hospital Anxiety and Depression Scale” (HADS) [22], “The Cervical Dysplasia Distress Questionnaire” (CDDQ) [23], and the specific “Process and Outcome Specific Measure” (POSM) scale [24] were used in this research. Depressiveness was defined as a cognitive triad, including a negative view of oneself, a negative view of the world, and a negative view of the future [25].

The CES-D scale (which contains twenty items) is a self-report depression scale, i.e., a screening test to identify persons who are at risk for depression [21]. Women were asked to indicate the level of agreement with each item, which were scored on a 4-point scale ranging from 0 to 3; thus, the CES-D scale total possible score ranges from 0 to 60. In this study, a score of ≥16 was taken as indicative of depression.

The CDDQ is a specific questionnaire that estimates psychological distress regarding cervical dysplasia [23]. This questionnaire has 23 questions and 4 domains; two domains, “Tension and discomfort” and “Embarrassment”, measure psychological distress associated with medical procedures (colposcopy), while two domains, “Concerns about sexual and reproductive consequences” and “Concerns about health consequences”, measure the psychological distress associated with the consequences of receiving an abnormal Pap smear result.

The HADS is a self-completion tool for identifying and quantifying anxiety and depression [22]. The HADS has 14 items that make up two subscales: 7 items relating to anxiety and 7 items relating to depression, within a week of the data collection. In this study, a score of 8–21 denoted presence of anxiety/depressive symptoms.

POSM is a specific scale that estimates the psychosocial burden among those women who received a positive cervical cancer screening result [24]. The scale comprises two factors (i.e., “Worry” (which contains four items) and “Satisfaction with information/support” (which contains three items) [24]. Prior to the start of this research, the Serbian versions of all used measurement tools had their validity and reliability confirmed, based on the internationally accepted methodology [26,27,28,29].

### 2.7. Ethical Considerations

This study was part of research approved by the Ethics Committee of the Faculty of Medical Sciences, University of Kragujevac (Ref. No.: 01-2176) and by the Ethics Committee of the Clinical Center Kragujevac (Ref. No.: 01-2869). All participants provided written informed voluntary consent prior to taking part in the study and confidentiality was protected.

### 2.8. Statistical Analysis

Methods of descriptive statistics were used to present categorical variables as absolute numbers and frequencies, and the chi-squared test was used to compare these variables. The ANN model that was used in this study was the multilayered perceptron, which uses a supervised learning technique algorithm carried out through feedforward back-propagation. The investigated outcomes were absence or presence of pre-diagnostic and post-diagnostic depression (labeled as 1 for CES-D scores < 16 and labeled as 2 for CES-D scores ≥ 16). Entry variables were sociodemographic and epidemiological characteristics of participants. Feature selection was performed using the filter method of correlation-based attribute selection, alongside the ranker search method. Variables that contribute most to the prediction model were chosen by estimating the Pearson correlation between the attribute and the class. The dataset was split using the unsupervised resample filter in order to create random dataset subsamples of 60% of the dataset for training, 20% for validation, and 20% for testing (external validation). The 10-fold cross-validation with “trial & error” method was used to construct the model and select the best parameters. The confusion matrix and kappa statistic were used to evaluate models. Accuracy, rate of false positive and rate of false negative outcomes, precision, ROC curve, and Matthews correlation coefficient were indicators of model performance. TRIPOD+AI guidelines were followed (Table S1). All statistical analyses were carried out in SPSS (The Statistical Package for Social Sciences software, SPSS Inc, version 20.0, Chicago, IL, USA), while the model building was performed using the Waikato Environment for Knowledge Analysis program (Weka, version 3.8.0, Waikato, New Zealand). Statistical significance was considered for *p*-values < 0.05.

## 3. Results

Just over half (56.4%) of the women were aged ≤ 50 years (Table 1). Most participants had an urban residence, a higher level of educational level, and a partner. The frequency of symptoms of depression (according to the CES-D scale, with a score ≥ 16) was higher prior to diagnostic procedures (36.6%) than after diagnostics (32.0%); the difference in prevalence of depression prior to (36.6%) and following the diagnostics (32.0%) did not show statistical significance, but the mean difference (13.98 vs. 12.74) was statistically significant (*p* = 0.025). Following the diagnostic procedures, women with a positive Pap screening result who had depression were mostly in their fifth and sixth decade of life, but without reaching statistical significance (*p* = 0.056).

**Figure 1:**
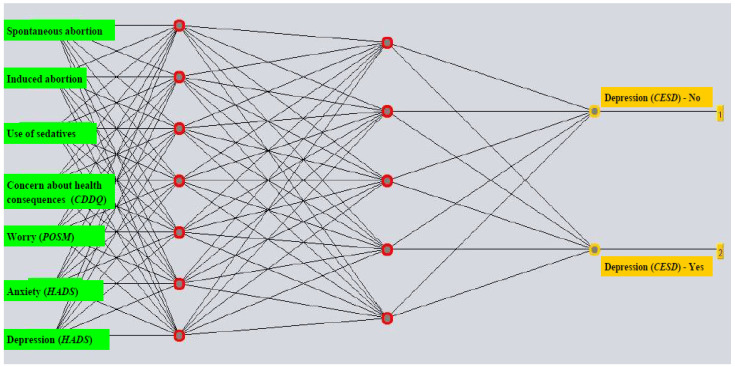
The multilayer perceptron structure of the model with the most significant attributes selected for predicting depression (according to the CES-D) among women with a positive Papanicolaou result prior to diagnostic procedures. Abbreviations: HADS (Hospital Anxiety and Depression Scale); CDDQ (Cervical Dysplasia Distress Questionnaire); CES-D (the Center for Epidemiologic Studies Depression); POSM (Process and Outcome Specific Measure).

**Figure 2:**
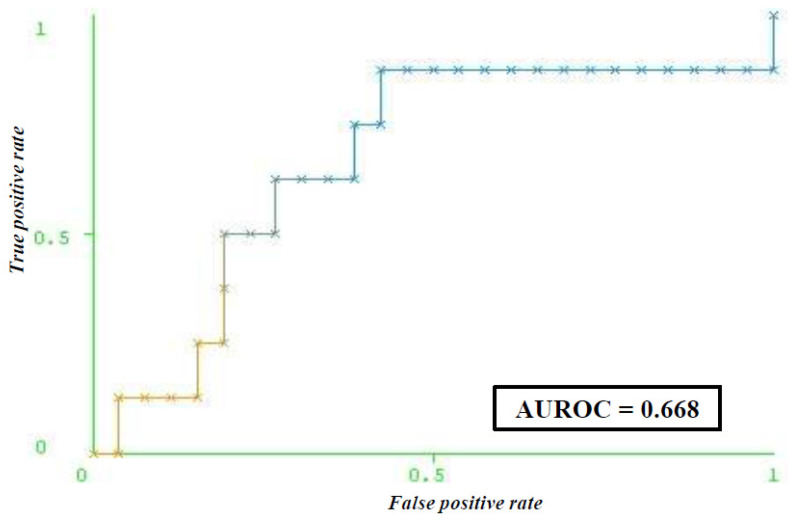
ROC curve for predicting depression (according to the CES-D scale) among women with a positive Papanicolaou result prior to diagnostic procedures. Abbreviation: CES-D (the Center for Epidemiologic Studies Depression); AUROC (The area under the receiver operating characteristic curve).

The ANN model for predicting pre-diagnostic depression with all attributes included was built with a learning rate of 0.4, a momentum of 0.6, 1000 epochs, and half of the sum of attributes and classes as the number of neurons in the hidden layer. The model with selected attributes had the following parameters: a learning rate of 0.4, a momentum of 0.6, 1000 epochs, 7 neurons in the first hidden layer, and 5 neurons in the second hidden layer (Figure 1). Attribute selection using correlation of attributes with the predicted class yielded seven variables relevant for predicting pre-diagnostic depression: HADS depression score, HADS anxiety score, POSM Worry score, CDDQ score for concern about health consequences, use of sedatives, induced abortion, and spontaneous abortion (Table 2). Selecting attributes that correlate with the class (predicted outcome) enabled the creation of a model with higher accuracy compared to that with the entire set of attributes. The model for predicting pre-diagnostic depression had a sensitivity of 70.6% and a specificity of 47.7% (Table 3). The AUROC value that the model achieved was 0.668 (Figure 2).

**Figure 3:**
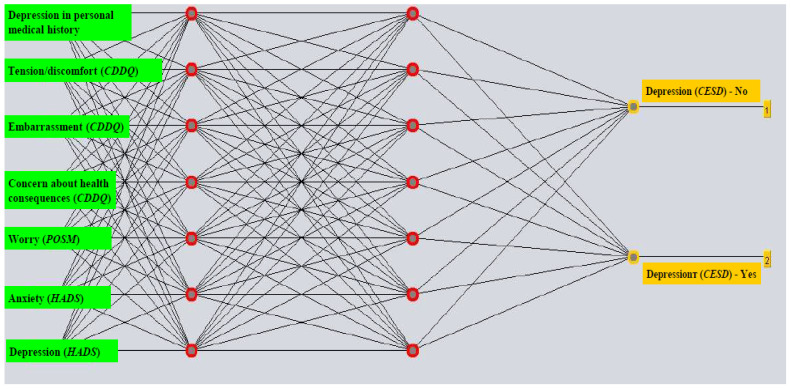
The multilayer perceptron structure of the model with the most significant attributes selected for predicting depression (according to the CES-D scale) among women with a positive Papanicolaou result following the diagnostic procedures. Abbreviations: HADS (Hospital Anxiety and Depression Scale); CDDQ (Cervical Dysplasia Distress Questionnaire); CES-D (the Center for Epidemiologic Studies Depression); POSM (Process and Outcome Specific Measure).

**Figure 4:**
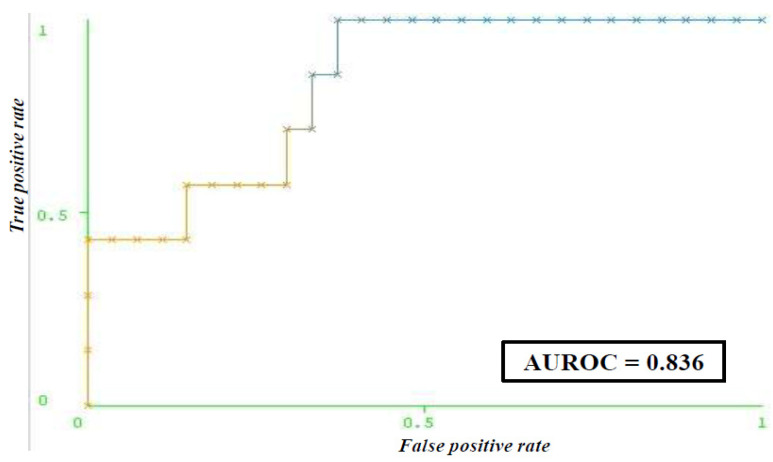
ROC curve for predicting depression (according to the CES-D scale) among women with a positive Papanicolaou result following the diagnostic procedures. Abbreviation: CES-D (the Center for Epidemiologic Studies Depression); AUROC (The area under the receiver operating characteristic curve).

Prediction of post-diagnostic depression in women with a positive Pap screening test employed an ANN model (with all attributes) with a learning rate of 0.6, a momentum of 0.3, 1000 epochs, and two hidden layers, with half of the sum of attributes and classes as the number of neurons in one hidden layer, and the sum of attributes and classes as the number of neurons in the other hidden layer. For the model with selected attributes, the parameters were: a learning rate of 0.6, a momentum of 0.1, 1000 epochs, and two hidden layers, each with 7 neurons (Figure 3). The selection of the most important features involved the HADS depression score, HADS anxiety score, POSM Worry score, CDDQ score for concern about health consequences, CDDQ score for Tension/discomfort, depression in personal medical history, and CDDQ score for Embarrassment (Table 2). The post-diagnostics depression prediction model showed a sensitivity and a specificity of 70.6% and 72.2%, respectively (Table 4). An area under the ROC curve score of 0.836 indicated that the created model is a good classifier (Figure 4).

## 4. Discussion

The data regarding the experiences of women who had an abnormal Pap screening result and underwent further diagnostics is scarce in Serbia. To the best of our knowledge, in the available literature, there are no studies that investigated the level of depression prior to and after diagnostics in women who received a positive Pap screening test result, nor are there studies about the application of ANNs for predicting pre- and post-diagnostic depression in this population.

A systematic review of adverse psychological outcomes following colposcopy and related procedures found great heterogeneity between the studies and outlined that pre- and post-colposcopy experiences of depression varied among women as an adverse response to their abnormal cytology test result [17]. Some authors reported that women who had an abnormal Pap smear and were waiting for colposcopy did not experience depression [30,31]. Also, some research in women who were undergoing colposcopy showed no differences either in the prevalence or in the risk of depression weeks after the procedure [20] or found a small change in average depression scores between the initial visit and later follow-up [19]. Our findings are consistent with the results obtained in these studies. The observed higher mean post-colposcopy CES-D score aside, this research indicates that undergoing colposcopy and other diagnostics does not have an effect on the frequency of depression, which might be due to these exhibiting no effects on depression, or due to having the information/support that is given to these women successfully alleviate adverse psychosocial consequences, or possibly accepting and undergoing diagnostic procedures can to some extent suppress depression which happens following the receipt of an abnormal Pap result [13,32].

It is known that prior to and following diagnostic procedures in women with a positive Pap screening test, higher anxiety and a higher degree of worry according to the POSM scale represent significant correlates of depression [33], a finding that was also confirmed in our study. For women, having a positive Pap result and undergoing follow-up diagnostics can lead to increased stress, painful diagnostic and therapeutic procedures, fear for future offspring, and feelings of self-blame, as well as dissatisfaction with the support of the environment [34]. It is possible that some other psychosocial problems are causing the increased distress, such as concerns regarding health, sexual life, ability to have children, fear of developing cervical cancer, or concerns about body image [35,36,37,38]. An unfavorable score for the mental component of quality of life and marked anxiety were significantly more common after colposcopy [39]. In addition, women with positive colposcopy-based cervical cytology had significantly lower values for the sleep and sexual activity dimensions in comparison with the general population [40].

In the present study, depression in personal medical history was selected as an attribute that correlates with depression after diagnostic procedures. In contrast, in a study of Latin American Caribbean women who underwent colposcopy because they had an abnormal Pap smear, women who had a previous diagnosis of depression did not experience more stress in terms of the medical procedure of colposcopy than counterparts who were not so diagnosed [28]. Similar to the findings of Shinn et al. [23], our study indicated that the CDDQ score for concern about health consequences was selected as the attribute that correlates with depression both prior to and following diagnostic procedures, whereby the CDDQ scores for Tension/discomfort and for Embarrassment were selected as attributes that correlate with post-diagnostic depression. However, it remains a question whether the cause of depression in women might not be directly related to the colposcopy or the waiting time for the results, but may actually be related to some other events in their life, either before or after both the screening and the diagnostic procedures.

Similar to this research, some studies have documented the use of sedatives during cervical cancer screening and receipt of abnormal Pap test results [41], as well as among women diagnosed with cervical cancer [42]. The correlation between sedative use and post-diagnostic depression that was recorded in this research could involve being scared of cancer and/or fearing the unknown while waiting for the results of follow-up procedures, insufficient support from the people around them, and not having enough information about what an abnormal Pap result actually means, and could also be due to the fact that the organized screening program for cervical cancer has only recently been implemented in Serbia.

To date, there are no available reports regarding the association between depression and a history of either spontaneous or induced abortion among women with a positive Pap result prior to diagnostics. It is known that abortion abruptly ends the maternal biological hormonal process that lasts during pregnancy, which can lead to somatic and psychological consequences, including post-traumatic stress, anxiety, and depression [43,44]. Globally, the overall pooled prevalence of post-abortion depression was reported to be 34.5%, while the highest frequency (42.91%) was found in lower-middle-income countries [45]. A study in the United Kingdom reported that recurrent miscarriage and emergency contraception were more likely to occur in women who had a mental illness compared to those without, while they were also less likely to take part in cervical cancer screening [46]. Conversely, having a history of abortion significantly correlated with a higher chance of attending cervical cancer screening in women of reproductive age in Kenya [47]. Still, caution is necessary in interpreting this correlation. The correlation between pre-diagnostic depression and abortion could be due to women who had an abortion having more frequent interactions with healthcare services, and thus having a better awareness of cervical cancer screening. Finally, it cannot be fully excluded that it is possible that recall bias exists on this matter, since it is possible that women who experienced certain troubles were more willing to recall abortion and other health-related events.

### Strengths and Limitations of the Research

To the best of our knowledge, there are not many studies in the available literature worldwide that have examined pre- and post-diagnostic depression in women who received a positive Pap screening smear. Based on the available literature, so far no research has been carried out that involved the use of ANNs among women who received an abnormal Pap screening test. A further strength of this study is that it only used validated questionnaires [22,23,24,25]. However, this study has several potential sources of limitations. These include the inherent shortcomings of the applied cross-sectional study design, the use of self-report questionnaires, the risk of information bias, the issue of the size of the study sample, the issue of the representativeness of the study sample, the lack of assessment of the level of depression before the Papanicolaou test, the absence of clinical confirmation of depression during the study, absence of insight into the medical documentation of the subjects in the study, lack of data on some other characteristics of the respondents in the research (such as HPV status, socio-economic status, etc.), and also the impossibility of ruling out the effect of exposure to some other factors on the levels of pre- and post-diagnostic depression.

## 5. Conclusions

Women with an abnormal Papanicolaou test, both before and after additional diagnostic procedures, experience a substantial level of depressive symptoms. This study created ANN models that could help in identifying which women with a positive Pap test are at risk for depression prior to and after the follow-up diagnostics. That way, women who take part in cervical cancer screening can receive psychosocial support throughout all of the procedures of the program, which could in turn improve screening coverage and significantly improve survival.

## Figures and Tables

**Table 1:** Women (N = 172) with abnormal Papanicolaou smear results: sociodemographic characteristics and prevalence of depression before and after diagnostic procedures.

Variables		Depression—Present			
	Total	Before Diagnostic Procedures		After Diagnostic Procedures	
	Number (%)	Number (%)	*p* *	Number (%)	*p* *^,^**
Age (years)					
- ≤30	12 (7.0)	6 (9.5)		7 (12.7)	
- 31–40	43 (25.0)	12 (19.0)		8 (14.5)	
- 41–50	42 (24.4)	18 (28.6)		17 (30.9)	
- 51–60	51 (29.7)	18 (28.6)		14 (25.5)	
- ≥61	24 (14.0)	9 (14.3)	0.582	9 (16.4)	0.056 *
Place of residence					
- Rural	45 (26.2)	21 (33.3)		18 (32.7)	
- Urban	127 (73.8)	42 (66.7)	0.155	37 (67.3)	0.268
Education level					
- ≤8 years	37 (21.5)	11 (17.5)		11 (20.0)	
- >8 years	135 (78.5)	52 (82.5)	0.441	44 (80.0)	0.843
Marital status					
- Without partner	33 (19.2)	13 (20.6)		11 (20.0)	
- With partner	139 (80.8)	50 (79.4)	0.841	44 (80.0)	0.838
Depression (CES-D score ≥ 16)					
- No		109 (63.4)		117 (68.0)	
- Yes		63 (36.6)		55 (32.0)	0.364 *
Mean ± Standard Deviation		13.98 ± 9.56		12.74 ± 9.15	0.025 **
Range		0–43		0–38	

Abbreviation: CES-D (the Center for Epidemiologic Studies Depression). *p* (value by: * χ^2^-test, ** Wilcoxon rank test).

**Table 2:** Correlation-based feature selection for predicting depression (according to the CES-D scale) in women with a positive Papanicolaou result prior to and following the diagnostic procedures.

Attributes	Depression—Before Depression—After	
	Pearson Correlation Coefficient	
HADS depression score	0.52741	0.41952
HADS anxiety score	0.46719	0.42443
POSM Worry score	0.29532	0.29432
CDDQ score for concern about health consequences	0.21773	0.20956
Use of sedatives	0.24591	
Induced abortion	0.21501	
Spontaneous abortion	0.20992	
CDDQ score for Tension/discomfort		0.25198
Depression in personal medical history		0.22991
CDDQ score for Embarrassment		0.21929

Abbreviations: CES-D (the Center for Epidemiologic Studies Depression); HADS (Hospital Anxiety and Depression Scale); CDDQ (Cervical Dysplasia Distress Questionnaire); POSM (Process and Outcome Specific Measure).

**Table 3:** Metrics for evaluating the classification model for predicting depression (according to the CES-D scale) among women with a positive Papanicolaou result prior to diagnostic procedures.

Evaluation Metrics	Model: Training + Validation Set ** with All Attributes	Model: Test Set with All Attributes	Model: Training + Validation Set ** with Selected Attributes	Model: Test Set with Selected Attributes
Accuracy	71.0145%	64.7059%	71.7391%	70.5882%
Kappa	0.395	0.097	0.419	0.182
TP Rate *	0.710	0.647	0.717	0.706
FP Rate *	0.315	0.541	0.292	0.523
Precision * (PPV)	0.710	0.676	0.723	0.706
NPV	0.555	0.300	0.633	0.375
ROC Area *	0.787	0.572	0.762	0.668
MCC	0.395	0.098	0.421	0.183

Abbreviations: CES-D (the Center for Epidemiologic Studies Depression); TP (True Positive rate); FP (False Positive rate); PPV (Positive Predictive Value); NPV (Negative Predictive Value); ROC (the receiver operating characteristic curve); MCC (Matthews’ correlation coefficient). * pondered arithmetic mean for both classes; ** 10-fold cross validation.

**Table 4:** Metrics for evaluating the classification model for predicting depression (according to the CES-D scale) among women with a positive Papanicolaou result following the diagnostic procedures.

Evaluation Metrics	Model: Training + Validation Set ** with All Attributes	Model: Test Set with All Attributes	Model: Training + Validation Set ** with Selected Attributes	Model: Test Set with Selected Attributes
Accuracy	68.1159%	58.8235%	72.4638%	70.5882%
Kappa	0.310	0.044	0.415	0.317
TP Rate *	0.681	0.588	0.725	0.706
FP Rate *	0.364	0.530	0.293	0.288
Precision * (PPV)	0.688	0.690	0.738	0.798
NPV	0.538	0.230	0.589	0.384
ROC Area *	0.680	0.593	0.744	0.836
MCC	0.311	0.048	0.419	0.348

Abbreviations: CES-D (the Center for Epidemiologic Studies Depression); TP (True Positive rate); FP (False Positive rate); PPV (Positive Predictive Value); NPV (Negative Predictive Value); ROC (the receiver operating characteristic curve); MCC (Matthews’ correlation coefficient). * pondered arithmetic mean for both classes; ** 10-fold cross validation.

## Data Availability

All data are contained within the article.

## Supplementary Materials

The following supporting information can be downloaded at: https://www.mdpi.com/article/10.3390/life14091130/s1, Table S1: TRIPOD+AI Checklist [48,49].
